# Association between Swallowing-Related Questionnaire Responses and Pathological Signs on Videofluoroscopy in Children

**DOI:** 10.3390/children8121109

**Published:** 2021-12-01

**Authors:** Jana Jančíková, Denisa Bezděková, Petra Urbanová, Lucie Dohnalová, Petr Jabandžiev, Miroslav Tedla, Žofia Frajková, Jiří Jarkovský, Milan Urík

**Affiliations:** 1 Department of Pediatric Otorhinolaryngology, University Hospital Brno and Faculty of Medicine, Masaryk University Brno, 61300 Brno, Czech Republic; jancikova.jana@fnbrno.cz (J.J.); bezdekova.denisa@fnbrno.cz (D.B.); 2 Department of Pediatric Radiology, University Hospital Brno and Faculty of Medicine, Masaryk University Brno, 61300 Brno, Czech Republic; urbanova.petra@fnbrno.cz (P.U.); dohnalova.lucie@fnbrno.cz (L.D.); 3 Department of Pediatrics, University Hospital Brno and Faculty of Medicine, Masaryk University Brno, 61300 Brno, Czech Republic; jabandziev.petr@fnbrno.cz; 4 Department of Otolaryngology, Head and Neck Surgery, Comenius University, University Hospital, 85107 Bratislava, Slovakia; miro.tedla@gmail.com (M.T.); zofia.frajkova@gmail.com (Ž.F.); 5 Institute of Biostatistics and Analyses, Faculty of Medicine, Masaryk University Brno, 62500 Brno, Czech Republic; jarkovsky@iba.muni.cz

**Keywords:** dysphagia, children, specific questionnaire, videofluoroscopy, aspiration

## Abstract

The aim of this study was to identify relationships in children between responses to specific questions of interest in a clinical questionnaire concerning swallowing-related difficulties and pathological signs on a videofluoroscopic swallowing study (VFSS). A prospective data analysis was made of children evaluated with swallowing disorder between January 2018 and April 2021 at a tertiary care centre. Each child enrolled in the study underwent a subjective evaluation (targeted questions) and instrumental examination (VFSS). In total, 51 children suffering from swallowing problems (32 with a neurological disorder and 19 without neurological disorder) were included into the study. Our results showed there was a correlation between the occurrence of specific symptoms (wet voice, wet breathing, recurrent respiratory infections, chronic mucus) and other pathological signs on a VFSS (laryngeal penetration, residua, nasal regurgitation). The evaluation of these specific questions is a reliable and useful method for the management of dysphagia in neonates and infants. It can help us in selecting those patients for which it is appropriate to perform a VFSS.

## 1. Introduction

Suspicion of aspiration is the main reason for instrumental swallowing evaluation in children [1,2]. Clinical symptoms of aspiration typically include recurrent infections of the airways, wet voice, wet breathing, coughing and/or choking, apnoea, and chronic mucus in the airways [3]. Although instrumental assessments are well-validated, there is limited information available to guide the selection and use of non-instrumental assessments for swallowing and feeding function in children. Little is known about the correlation between clinical symptoms based on the evaluation of specific questions of interest and the probability of finding aspiration or other pathological signs by videofluoroscopic swallowing study (VFSS) [3,4].

The subjective evaluation of swallowing typically consists of a series of questions, and it is completed by a speech-language pathologist (SLP) specialized in the treatment of children with dysphagia in cooperation with the parent and/or legal guardian of the child. It also typically includes an observed feeding at the bedside.

Instrumental swallowing assessment is crucial in children with chronic respiratory symptoms because typical signs of aspiration, such as aspiration pneumonia, are rare, occurring in less than 10% of children [5,6]. The visualization of swallowing in the paediatric population is performed during a VFSS or flexible endoscopic evaluation of swallowing (FEES). These methods are complementary and have their advantages and disadvantages [7]. A VFSS is a comprehensive evaluation that provides dynamic imaging of the swallowing phases. Common abnormalities identified during a VFSS include pooling, residua, laryngeal penetration, and aspiration [8]. Choosing the most appropriate combination of subjective and objective assessment tools is key to early diagnosis and treatment of paediatric dysphagia [9,10]. Inadequately treated dysphagia and aspiration can lead to a variety of poor outcomes, including lung damage, failure to thrive [11], and oral aversion [12,13,14].

The aim of this study was to identify the relationships between responses to the specific questions (SQ) in a questionnaire and pathological signs on a VFSS in children. Second, we compared the questionnaire outcomes between two groups of children—those with and without neurological disability—because it is well known that neurological disorders are associated with oropharyngeal dysphagia in children [15]. The originality of this study lies in its prospective data collection while using the targeted SQ in children without age restrictions.

## 2. Materials and Methods

### 2.1. Data Collection

The prospective data analysis and evaluation were performed as part of routine clinical procedures between January 2018 and April 2021 at a tertiary care centre. The study protocol was approved by the ethics committee of University Hospital Brno (No. 11-101121/EK). Informed consent of the parent and/or legal guardian was given before intervention. We examined 117 children with swallowing problems during this period. All children underwent an examination by an ear, nose and throat doctor and an SLP, and for all children we completed the SQ questionnaire. Taken as absolute indication for VFSS were clinical signs of aspiration, which means the presence of respiratory symptoms related to eating or aspiration pneumonia in the patient history. Relative criteria included other problems in the oropharyngeal phase of the swallowing cycle (as summarized in the questionnaire). The questions were not asked on the same day as the VFSS, the time between these two assessments being about 2–3 weeks on average.

### 2.2. Procedure

All children underwent a clinical swallowing and feeding examination by an SLP and the SQ questionnaire was evaluated in cooperation with a parent and/or legal guardian or with nurses if a child was in hospital without a parent and/or legal guardian. The questionnaire for parents consisted of the following questions: has your child recurrent respiratory infections or long-term airway obstruction, voice changes after eating (wet voice, wet breathing, “gurgling”), cough during eating, weight stagnation, failure to thrive, weight loss, solid food intolerance, abundant salivation during the day, or difficulty in drinking fluids?

The VFSS was conducted by a multidisciplinary team composed of an SLP, a paediatric otorhinolaryngologist specializing in paediatric swallowing disorders, and a radiologist. The VFSS procedures were performed in a lateral projection using a standard diagnostic protocol. Newborns and toddlers were examined sitting in a wooden car seat (no metal parts). Older children were sitting in a chair or standing during the assessment. Various consistencies of solids and liquids containing barium (Micropaque solution, Guerbet, Villepinte, France) were used. The types of solids and liquids tested during the VFSS depended upon a child’s age and results of the clinical evaluation of feeding and swallowing. It is our standard practice during a paediatric VFSS to offer three teaspoons of a solid consistency (e.g., a biscuit), then three sips of pudding consistency (e.g., a fruit puree or pudding), and finally three sips of any liquid from a cup or bottle (tea, milk or water, or barium suspension only).

### 2.3. Oropharyngeal Transit Time

Oral transit time (OTT) is the time (measured in seconds) from the onset of bolus movement in the mouth until the reflex firing of swallowing. Pharyngeal transit time (PTT) starts when the food bolus is in the hypopharynx (at the point where the inferior border of the lower jaw makes an angle with the base of the tongue), and it ends when the food bolus has passed through the upper oesophageal sphincter. We measured the oropharyngeal transit time (OPTT) as the sum of OTT + PTT. Specialized radiological software was used for the quantitative analysis that enabled the time to be registered in seconds.

The eight-point Penetration–Aspiration Scale described by Rosenbek et al. was used to evaluate penetration and aspiration [16]. That scale was developed to describe the degree of laryngeal penetration and aspiration as well as the patient’s response.

### 2.4. Statistical Analysis

Absolute and relative frequencies are given for categorical variables. Continuous variables are described using the median in combination with the 25th and 75th percentiles. The *p*-values of Fisher’s exact test are given for categorical variables and the *p*-values of the Mann–Whitney test for continuous variables.

## 3. Results

Of the 117 subjects that were screened, 51 were deemed high risk and underwent a VFSS and screening with the specific questions of interest. In total, 51 children suffering from swallowing problems (32 with a neurological disorder and 19 without neurological disorder) were included into the study. This cohort comprised 24 females and 27 males. The mean age of patients was 17.8 (±15.6) months, ranging from the youngest, at 21 days, up to 62 months of age.

A high risk of laryngeal penetration on a VFSS in children was identified by the presence of wet voice, wet breathing, recurrent respiratory infections (RRI) and/or chronic mucus in the airway reported during subjective evaluation (Table 1). A high risk of aspiration was accompanied by very similar clinical signs, with VFSS associating this risk in children with the presence of wet breathing, RRI, and chronic mucus in the airway. Aspiration occurred in 14 (27.5%) and penetration in 25 (49%) of the patients. The risk of nasal regurgitation on the VFSS was higher in children with RRI and wet breathing, and the risk of residua was higher in children with RRI and chronic mucus. All data are summarized in Table 2, Table 3 and Table 4.

High values on the Penetration–Aspiration Scale (PAS) were observed to be associated with the presence of wet voice, wet breathing, RRI, and chronic mucus (Table 5). Long oropharyngeal transit time (OPTT) values were also seen to be associated with the presence of wet voice, wet breathing, RRI, and chronic mucus (Table 6). We found a long OPTT value to be also associated with a higher risk of aspiration on the VFSS (*p* < 0.001, Mann–Whitney test). There were generally no differences in the occurrence of SQ symptoms across individual age categories (0–6 months, 7–12 months, and older than 12 months). The only exception was that nasal regurgitation occurred more frequently in the category 0–6 months (*p* = 0.033, Fisher’s exact test) (Table 7).

The statistical analysis showed that only RRI occurred more frequently in children with neurological disability than in children without neurological disability (*p* = 0.033, Fisher’s exact test). The occurrence of other SQ symptoms was very similar in the two groups. Upon the VFSS, however, we identified many more differences between these two groups. Greater occurrences of residua (*p* = 0.003), penetration (*p* = 0.020), aspiration (*p* = 0.020), and nasal regurgitation (*p* < 0.001) were statistically significant in children with neurological disability, as were longer OTT (*p* < 0.001) and higher PAS value (*p* < 0.001) (Table 8).

## 4. Discussion

The primary aim of this study was to identify the relationships between the SQ responses and pathological signs on a VFSS in children.

As perinatal survival outcomes for premature and otherwise vulnerable neonates have improved with technological advances, a greater number of neonates are surviving with significant feeding difficulties [17]. To establish effective feeding is a prerequisite for the survival of neonates and infants. The diagnosis and management of dysphagia and aspiration in neonates is a significant challenge for physicians, with management requiring long-term medical supervision and attention. Without intervention, the patient may develop recurrent illness, inadequate nutrition, and a need for supplemental nutrition that, if continued for an extended period, may result in oral aversion and refusal behaviours [18]. Successful management begins with a thorough evaluation that includes a complete history, physical examination, and appropriate imaging studies to identify the aetiology and potential targets for intervention.

The initial assessment starts with a clinical examination by a speech-language pathologist. Detection of a wet voice or wet breathing and/or chest congestion and cough after taking liquids by mouth is often associated with thin fluid aspiration. Other research has demonstrated that findings of chest congestion or rattling after consuming 90 mL of water have a high sensitivity but poor specificity for aspiration. In our centre, we used a modified swallowing specific questionnaire that is completed by an SLP in cooperation with parents or nurses.

We had hypothesized that the signs specified in the SQ questionnaire would be associated with a high risk of aspiration on a VFSS. Although our hypothesis was not supported, we determined that an SQ questionnaire documenting RRI, wet breathing, and chronic mucus is associated with increased odds of aspiration on a VFSS.

Our results showed there was a correlation between the occurrence of specific symptoms (wet voice, wet breathing, RRI, chronic mucus) and other pathological signs on a VFSS (laryngeal penetration, residua, nasal regurgitation). These findings are very interesting because they indicate the importance of SQ in the screening of swallowing disabilities in neonates and infants. There have been some studies about correlation between clinical feeding evaluation and a VFSS [19,20,21], but in our study we present only an evaluation based upon specific questions.

The sensitivity of the clinical feeding evaluation compared to a VFSS ranges from 33% to 92%, and it is particularly low for silent aspiration, which is present in 81% of children with aspiration. This suggests that clinical feeding evaluation alone may be inadequate to diagnose aspiration in this population [22,23,24]. We showed that the occurrence of clinical signs such as cough, wet voice, wet breathing, RRI, and chronic mucus in patients with aspiration on a VFSS ranges from 50% to 93%.

The secondary aim of our study was to compare the questionnaire outcomes between two groups of children—those with and without neurological disability.

Oropharyngeal dysphagia is an extremely common digestive disorder amongst children with neurological disorders and cerebral palsy, with reported prevalence exceeding 90% [25,26]. The main complications of oropharyngeal dysphagia include respiratory infections, aspiration pneumonia, and dehydration [27,28].

In our study, we found more frequent respiratory infections and a higher incidence of pathological signs on a VFSS in children with neurological disabilities compared to children without neurological disabilities. Children with neurological disability have respiratory infections more frequently than do children without neurological disability, as well as a higher incidence of pathological signs on a VFSS.

The limitation of this study is that there is the possibility of some selection bias since not all 117 underwent the complete evaluation and so it is possible that the combination of clinical feeding evaluation and specific questions might be required in some patients.

## 5. Conclusions

Many non-instrumental assessments are available to clinicians for evaluating swallowing and feeding function in paediatric populations. Evaluation of the specific questions is a reliable and useful method in the management of dysphagia in neonates and infants. It can help us in selecting patients for which a VFSS is appropriate.

## Figures and Tables

**Table 1:** Relationships between clinical signs and occurrence of laryngeal penetration in videofluoroscopic swallowing study.

Clinical Sign	Laryngeal Penetration		*p*-Value **
	Yes N = 25 *	No N = 26 *	
Cough	15 (60.0%)	10 (38.5%)	0.165
RRI	14 (56.0%)	5 (19.2%)	0.009
Wet voice	15 (60.0%)	4 (15.4%)	0.001
Wet breathing	17 (68.0%)	5 (19.2%)	0.001
Chronic mucus	20 (80.0%)	13 (50.0%)	0.040

* Absolute and relative frequencies are given for clinical signs. ** *p*-value of Fisher’s exact test. RRI—recurrent respiratory infections.

**Table 2:** Relationships between clinical signs and occurrence of residua in videofluoroscopic swallowing study.

Clinical Sign	Residua		*p*-Value **
	Yes N = 25 *	No N = 26 *	
Cough	10 (40.0%)	15 (57.7%)	0.267
RRI	15 (60.0%)	4 (15.4%)	0.001
Wet voice	11 (44.0%)	8 (30.8%)	0.393
Wet breathing	12 (48.0%)	10 (38.5%)	0.577
Chronic mucus	20 (80.0%)	13 (50.0%)	0.040

* Absolute and relative frequencies are given for clinical signs. ** *p*-value of Fisher’s exact test. RRI—recurrent respiratory infections.

**Table 3:** Relationships between clinical signs and occurrence of aspiration in videofluoroscopic swallowing study.

Clinical Sign	Aspiration		*p*-Value **
	Yes N = 14 *	No N = 37 *	
Cough	7 (50.0%)	18 (48.6%)	1.000
RRI	11 (78.6%)	8 (21.6%)	<0.001
Wet voice	8 (57.1%)	11 (29.7%)	0.106
Wet breathing	10 (71.4%)	12 (32.4%)	0.025
Chronic mucus	13 (92.9%)	20 (54.1%)	0.010

* Absolute and relative frequencies are given for clinical signs. ** *p*-value of Fisher’s exact test. RRI—recurrent respiratory infections.

**Table 4:** Relationships between clinical signs and occurrence of nasopharyngeal penetration in videofluoroscopic swallowing study.

Clinical Sign	Nasopharyngeal Penetration		*p*-Value **
	Yes N = 27 *	No N = 24 *	
Cough	14 (51.9%)	11 (45.8%)	0.781
RRI	14 (51.9%)	5 (20.8%)	0.041
Wet voice	13 (48.1%)	6 (25.0%)	0.146
Wet breathing	17 (63.0%)	5 (20.8%)	0.004
Chronic mucus	20 (74.1%)	13 (54.2%)	0.156

* Absolute and relative frequencies are given for clinical signs. ** *p*-value of Fisher’s exact test. RRI—recurrent respiratory infections.

**Table 5:** Relationship between clinical signs and Penetration–Aspiration Scale (PAS) on VFSS.

Clinical Sign *		PAS **	*p*-Value ***
Cough	yes, N = 25 (49.0%)	3 (1; 6)	0.329
	no, N = 26 (51.0%)	2 (1; 6)	
RRI	yes, N = 19 (37.3%)	7 (2; 7)	0.001
	no, N = 32 (62.7%)	1 (1; 4)	
Wet voice	yes, N = 19 (37.3%)	5 (2; 7)	0.004
	no, N= 32 (62.7%)	1 (1; 4)	
Wet breathing	yes, N = 22 (43.1%)	5 (2; 7)	<0.001
	no, N = 29 (56.9%)	1 (1; 2)	
Chronic mucus	yes, N = 33 (64.7%)	4 (1; 7)	0.007
	no, N = 18 (35.3%)	1 (1; 4)	

* For clinical signs, absolute and relative frequencies from the total data set are given (N_total_ = 51). ** Median with 25th and 75th percentiles. *** *p*-value of Mann–Whitney test.

**Table 6:** Relationship between clinical signs and oropharyngeal transit time (OPTT).

Clinical Sign *		OPTT ** (Seconds)	*p*-Value ***
Cough	yes, N = 25 (49.0%)	2.0 (1.2; 2.4)	0.651
	no, N = 26 (51.0%)	2.0 (1.1; 2.7)	
RRI	yes, N = 19 (37.3%)	2.5 (2.0; 3.1)	<0.001
	no, N = 32 (62.7%)	1.4 (1.1; 2.1)	
Wet voice	yes, N = 19 (37.3%)	2.2 (2.0; 2.9)	0.027
	no, N= 32 (62.7%)	1.6 (1.1; 2.4)	
Wet breathing	yes, N = 22 (43.1%)	2.3 (2.0; 3.0)	0.002
	no, N = 29 (56.9%)	1.5 (1.1; 2.0)	
Chronic mucus	yes, N = 33 (64.7%)	2.2 (1.8; 2.9)	0.002
	no, N = 18 (35.3%)	1.2 (1.1; 2.0)	

* For clinical signs, absolute and relative frequencies from the total data set are given (N_total_ = 51). ** Median with 25th and 75th percentiles. *** *p*–value of Mann–Whitney test.

**Table 7:** Incidence of clinical signs depending on age.

Clinical Sign	0–6 Months N = 14 *	7–12 Months N = 12 *	Older Than 12 Months N = 25 *	*p* **
Cough	8 (57.1%)	7 (58.3%)	10 (40.0%)	0.511
RRI	6 (42.9%)	3 (25.0%)	10 (40.0%)	0.652
Wet voice	7 (50.0%)	3 (25.0%)	9 (36.0%)	0.448
Wet breathing	8 (57.1%)	5 (41.7%)	9 (36.0%)	0.506
Chronic mucus	9 (64.3%)	9 (75.0%)	15 (60.0%)	0.692
Residua	4 (28.6%)	6 (50.0%)	15 (60.0%)	0.194
Penetration	9 (64.3%)	7 (58.3%)	9 (36.0%)	0.219
Aspiration	5 (35.7%)	3 (25.0%)	6 (24.0%)	0.716
Nasopharyngeal penetration	11 (78.6%)	7 (58.3%)	9 (36.0%)	0.033
PAS (scale of 1–8)	4 (2; 7)	3 (1; 6)	2 (1; 5)	0.363
OPTT (seconds)	2.20 (1.80; 2.90)	1.20 (1.05; 2.50)	2.00 (1.20; 2.30)	0.208

* Categorical variables are described by absolute and relative frequency. Continuous variables are described by median with 25th and 75th percentiles. ** *p*-value of Fisher’s exact test for categorical variables and *p*-value of Kruskal–Wallis test for continuous variables. RRI—recurrent respiratory infections, PAS—Penetration–Aspiration Scale, OPTT—oropharyngeal transit time.

**Table 8:** Incidence of clinical signs in groups with and without neurological disability.

Clinical Sign	With Neurological Disability N = 32 *	Without Neurological Disability N = 19 *	*p* **
Cough	16 (50.0%)	9 (47.4%)	1.000
RRI	17 (53.1%)	2 (10.5%)	0.003
Wet voice	14 (43.8%)	5 (26.3%)	0.247
Wet breathing	16 (50.0%)	6 (31.6%)	0.250
Chronic mucus	24 (75.0%)	9 (47.4%)	0.070
Residua	21 (65.6%)	4 (21.1%)	0.003
Penetration	20 (62.5%)	5 (26.3%)	0.020
Aspiration	14 (43.8%)	0 (0.0%)	0.020
Nasopharyngeal penetration	25 (78.1%)	2 (10.5%)	<0.001
PAS (scale of 1–8)	5 (2; 7)	1 (1; 2)	<0.001
OPTT (seconds)	2.25 (1.83; 2.95)	1.20 (1.07; 1.80)	<0.001

* Absolute and relative frequencies are given for categorical variables. Continuous variables are described by median with 25th and 75th percentiles. ** *p*-value of Fisher’s exact test for categorical variables and the *p*-value of Mann–Whitney test for continuous variables. RRI—recurrent respiratory infections, PAS—Penetration–Aspiration Scale, OPTT—oropharyngeal transit time.

## Data Availability

The data presented in this study are available upon request from the corresponding author.
